# Effects of Racial Concordance and Perceived Racism on Emergency Department Patient Satisfaction

**DOI:** 10.5811/westjem.49041

**Published:** 2026-10-26

**Authors:** Rachel Gonzales, Margaret Wang, Charlotte Sagan, Sally Nijim, Anish Agarwal

**Affiliations:** * University of Pennsylvania, Perelman School of Medicine, Department of Emergency Medicine, Philadelphia, Pennsylvania; † University of Pennsylvania, Perelman School of Medicine, Center for Health Care Transformation and Innovation, Philadelphia, Pennsylvania; ‡ University of Pennsylvania, Perelman School of Medicine, Department of Family Medicine, Philadelphia, Pennsylvania; § University of Pennsylvania, Perelman School of Medicine, Philadelphia, Pennsylvania

## Abstract

**Introduction:**

Racial disparities in healthcare persist, with evidence suggesting that bias contributes to inequities in care and outcomes for Black patients. Racial concordance between patients and clinicians has been proposed as one strategy to improve patient satisfaction and health outcomes, but its impact in acute care settings such as the emergency department (ED) remains underexplored. The objective of this study was to assess how racial concordance between patient and clinician, together with patient perceptions of how race affected their care, influence patient satisfaction with care in the ED.

**Methods:**

This secondary analysis used data from a previously published text message–based survey of patients discharged from two EDs between August and September 2021. Patients were categorized as not completely discordant if at least one clinician (physician, advanced practice clinician, or nurse) shared their self-identified race. The primary outcome was patient ratings of experience with ED care (scored 0 = poor to 5 = excellent). Multivariable ordinal logistic regression was used to assess associations between racial concordance, perceived impact of race on care, and experience ratings.

**Results:**

Of 516 respondents, 195 (37.8%) were classified as not completely discordant with at least one emergency clinician. Not completely discordant patients reported significantly lower experience ratings (scored 0 to 5) compared to racially discordant patients (median, 4 [3–5] vs 5 [3–5]; *P* = .01). In multivariable ordinal logistic regression, racial concordance was not independently significant in patient experience.

**Conclusion:**

Contrary to expectations, in our population, racial concordance was not significantly associated with satisfaction in ED care. In the adjusted analysis, perceived negative impact on care due to race was the strongest independent predictor of lower overall satisfaction. These findings suggest that how patients perceived the role of race in their care may be more influential than simply patient-clinician concordance in the acute care setting. Further research is needed to investigate these factors in more diverse patient-clinician population and other ED settings to help inform policies focused on advancing health equity.

## INTRODUCTION

Racial disparities in healthcare have long persisted in the United States. Black Americans in particular experience disproportionately high burdens of chronic illness, maternal mortality, and mortality across many diseases.^1^–^4^ Experiences of racism in healthcare are associated with lower medication adherence, reduced participation in clinical research, and delays in seeking care.^5^–^7^ There is increasing evidence that racism contributes to these disparities, including inadvertently through implicit bias that can impact behaviors such as language used to describe patients in the electronic health record (EHR).^8^–^11^ Studies show that Black physicians do not demonstrate as much implicit racial preference as their non-Black colleagues.^12^,^13^

Racial concordance between clinician and patient may be protective against poor care and health outcomes as patients are more likely to use health services and less likely to delay seeking care;^14^ more likely to be satisfied with care;^15^ and more likely to start appropriate medication treatment.^16^ However, there is limited research that addresses race as a factor in patient satisfaction research and in acute care emergency department (ED) settings where interactions are brief, with fewer opportunities for a longitudinal relationship. Our recent study used a postdischarge survey to explicitly ask about experiences of racism in the ED, along with satisfaction with ED care.^17^ The data resulting from the survey were used in this secondary analysis. The objective of this secondary analysis was to explore how racial concordance between clinician and patient, together with patient perception of the role of race in their care, are associated with satisfaction with ED care.

## METHODS

Data and methodology are taken from our previously published data on ED care, and the data used for this analysis are a secondary analysis of the primary study.^17^ Briefly, discharged ED patients from two EDs within a large, urban academic health system in the Northeast were sent a text message-based survey to assess their perceptions of ED care. The eight-item survey was informed by existing literature on discrimination and racism in healthcare and drafted by the study team. which included experts in healthcare delivery, emergency care, and equity-based research. The survey included questions on satisfaction with ED care; the impact of race on care (positive, negative, or none); and whether race affected key components of care delivery (eg, respect, quality of service, and communication) (Figure S1).

This study represents a secondary analysis of this survey with linked ED-encounter EHR data. Whereas the primary paper restricted inclusion to patients who completed the entire survey, this analysis focused on participants who answered at least the question on ED experience rating. The primary outcome was satisfaction with ED care, examined in relation to racial concordance between clinician and patient and patient-reported effects of race on care. A patient was classified as “not completely discordant” with their clinician if they had the same self-identified race as at least one of their clinicians during their ED visit (attending or resident physician, advanced practice clinician [APC], or nurse) and racially discordant if they did not have the same race as any of their clinicians. Participant race collected from the EHR was grouped into four categories: White, Black, Asian, or other. Clinician race was determined by study team observations and knowledge and included both the first and last clinician seen in each role in the patient’s EHR. First and last clinician seen are defined as clinician providing direct care for a patient and thus responsible for the visit; the definition excludes clinician in triage. Ethnicity was not considered in the analysis. Statistical analyses were conducted using Stata SE 18.5 (StataCorp, LLC, College Station, TX) and Wilcoxon rank-sum tests to assess differences of medians in univariate analysis and ordinal logistic regression modeling.

**Population Health Research Capsule**

What do we already know about this issue?

*Racial disparities in healthcare persist; concordance may improve satisfaction, but emergency department (ED) evidence is limited and mixed.*

What was the research question?

*Does racial concordance and perceived race effects influence ED patient satisfaction?*

What was the major finding of the study?

*Negatively perceived race effect lowered satisfaction (odds ratio [OR] 0.16, P < .001).*

How does this improve population health?

*Targeting perceived bias, not just concordance, may better improve satisfaction and equity in ED care.*

## RESULTS

In a study of 516 patients discharged from an ED (August 6 and September 24, 2021, Figure S1), 195 (37.8%) patients were classified as not completely discordant, and 321 (62.2%) were racially discordant with all their clinicians (Table 1).

Several clinician types were seen at different rates between not completely discordant and completely discordant patients. Not completely discordant patients were more likely to have seen a physician (attending or resident) or nurse as part of their visit, whereas completely discordant patients were more likely to have seen an APC as part of their visit (50.2% vs 30.1%) (Table 1).

Not completely discordant patients had a *lower* median satisfaction rating with ED care compared to racially discordant patients (median, 4 [3–5] vs 5 [3–5]; *P* = .01) (Table 1), but no significant differences were found in responses to questions on respect, service quality, or communication; the significant difference observed in the median experience rating did not hold when looking at experience ratings as categorical variables. When stratified by type of clinician, only patients seen by a racially concordant attending physician continued to demonstrate a significant difference in the primary outcome (median, 4 [3–5] vs 5 [3–5]*; P* = .04). There was no significant difference in the distribution of answers on whether patients perceived race to have a positive, negative, or no effect on their care in the racially concordant vs discordant groups (*P* = .10) (Table 1).

When stratified by patient race, results of univariate analyses show no significant difference in median ratings of ED care between not completely discordant vs completely discordant groups for Black patients (4 [3–5] vs 5 [3–5], *P* = .08) or White patients (4 [3–5] vs 5 [4–5], *P* = .06) (Table S1). When stratified by patient report of how their race affected ED care, there was no significant difference in median experience ratings between not completely discordant vs discordant groups for patients reporting either a positive or negative effect. Among patients who reported that race had no effect on their care experience, the median rating was *lower* for not completely discordant patients (4 [3–5] vs 5 [5–5], *P* < .001).

Results of multivariable analyses using racial concordance and patient-reported effect of race on ED care showed that the only significant predictor affecting satisfaction rating were perceived negative effect of race on ED experience (odds ratio [OR] 0.16; 95% CI, 0.09–0.30; *P* < .001) and age (OR 1.01; 95% CI, 0.005–1.02*; P* = .03). Patients reporting race to have had a negative effect on their care had decreased odds of higher satisfaction ratings (Table 2). There was trend toward increased age leading to increased odds of higher satisfaction ratings as well.

## DISCUSSION

This study aimed to explore the relationship between racial concordance between patients and clinicians and satisfaction ratings of ED care. The strengths of this study include a relatively large and racially diverse patient sample and the ability to examine both concordance and perceived racial impact on care. The results demonstrate that the effects of racial concordance between patients and clinicians are complex and context-dependent. The study points to three main findings: (1) ED satisfaction ratings were not significantly different between patients who were classified as not completely discordant with their clinicians and those who were completely discordant in multivariate models; (2), patients who perceived their race to negatively affect their ED care rated their care lower, even when controlling for other variables; and (3) not completely discordant patients were more likely to be White than those who were completely discordant.

While racial concordance has been proposed as one strategy to improve healthcare equity, outcomes and experiences for Black patients, these findings suggest a complex relationship in the acute care setting that requires additional research. Specifically, we found that racial concordance alone was not a significant predictor of patient experience ratings in the ED settings. These findings align with emerging evidence that the effects of concordance are not universally positive or consistent across clinical settings.^18^–^21^ One meta-analysis found that while racial concordance was associated with improved communication, effects on patient satisfaction specifically were mixed—consistent with our finding that concordance alone does not uniformly predict satisfaction.^20^ A second ED-based study found no significant effect of racial concordance on patient perceptions of physician trust and respect, although age concordance showed a negative effect—paralleling our finding that the relationship between concordance and satisfaction may differ depending on clinical settings.^21^ Our analysis incorporated patients’ perceived experience of racial bias as a covariate, which emerged as a stronger predictor than concordance itself, underscoring the value of directly asking patients about race in care surveys.

Several factors may explain why racially concordant patients in our study reported lower satisfaction. The nature of ED care itself likely affected ratings. Unlike with longitudinal outpatient care, patients are not able to select for the demographics of a clinician, which may further influence their satisfaction of care. Prior research has shown that patient satisfaction may be influenced both by whether they receive care from a racially concordant clinician and by their perception of racial bias in healthcare.^15^ In multivariate analysis, patients who perceived race to have had a negative impact on their care were more likely to give lower satisfaction scores, while in stratified univariate analysis only those who were not completely discordant and did not perceive any impact of race on care indicated lower ratings than their discordant counterparts. While this latter finding may be in part due to a larger proportion of patients indicating a neutral perceived effect, together, these patterns suggest that perceived bias may be a stronger and more consistent determinant of satisfaction than concordance alone. Further, the relationship between concordance and experience may vary depending on patients’ racial identities and their expectations or interpretations of the encounter.

The racial composition of the not completely discordant group warrants consideration in interpreting these findings. Not completely discordant patients were more likely to be White than those who were completely racially discordant with their care team, which likely reflects the predominantly White racial composition of the workforce including across role type in the ED. White patients at these EDs were far more likely to share a race with at least one of their clinicians simply because more clinicians were White—meaning their not completely discordant classification may reflect structural workforce demographics rather than any meaningful patient-clinician relationship dynamic.

## LIMITATIONS

Limitations in this research include the lack of data on discordant White patient-clinician relationships, with only 7 White participants (2.2%) having had an encounter where no clinician on their care team was also White. This highlights an important area for future research to better understand the impact of racial concordance for all racial groups. Second, data were limited to one large academic center over a limited time frame, and the results may not be generalizable and reflect broader, overall ED care. Third, experience ratings were skewed, and the lowest categories contained few observations, resulting in a low events-per-parameter ratio in the ordinal regression. These small cell sizes may reduce the stability of the adjusted model estimates.

Fourth, our definition of racial concordance does not account for how much time each clinician spent with the patient, which was not collected as part of our study. This definition may overlook. clinicians who were not the first or last in their role but still interacted with the patient during the encounter. As a result, there may be substantial heterogeneity in who is classified as “racially concordant.” Selection bias may also be present given the discrepancy between those who received the survey and those who completed the study question. While the response rate (14.5%) is in line with response rates to other short message service-based surveys, it may reflect patient concerns about spam messages or characteristic differences between responders and non-responders that could not be assessed with this study’s data.

Additionally, we used first and last clinician abstracted from the EHR, excluding triage clinician, but we could not account for instances where additional clinicians were involved in a patient’s care. Finally, the robustness of key findings should be interpreted cautiously. The primary unadjusted result (*P* = .014) was derived from a Wilcoxon rank-sum test on the full ordinal scale; when satisfaction was dichotomized into low versus high ratings, the difference between NCD and discordant groups was no longer statistically significant (*P* = .12). This sensitivity analysis suggests the result was driven by subtle distributional differences across the rating scale rather than a large or robust group difference and underscores the need for replication in larger samples. Future directions include focusing on the relevance of racial concordance not only on experience data, but on patient health outcomes, both in an ED and in a longitudinal outpatient setting. Research is also needed to further explore the relationship between patient-clinician concordance and experience ratings in the ED.

## CONCLUSION

Including perception of racial bias on health surveys is an important metric affecting patient satisfaction. Furthermore, the presence of racial concordance itself is not a simple solution; instead, it is a complex yet significant predictor of patient satisfaction. More research on the interplay of these factors in diverse patient-clinician populations is needed to help inform policies focused on furthering health equity.

## Supplementary Information





## Figures and Tables

**Table 1:** Demographics of patients classified as not completely discordant versus complete racial discordance in a study to assess how racial concordance between patient and clinician, together with patient perceptions of how race affected their care, influence patient satisfaction with care in the emergency department.

	Not complete discordance (*n* = 195)	Complete racial discordance (*n* = 321)	*P* value
Sex, *n* (%)			.04
Female	124 (63.6)	216 (67.3)	
Male	69 (35.4)	91 (28.4)	
Missing	2 (1.0)	14 (4.4)	
Age, mean (SD)	45.0 (±18.4)	41.4 (±16.4)	.01
Race, *n* (%)			< .001
Black	43 (22.1)	277 (86.3)	
White	143 (73.3)	7 (2.2)	
Asian	9 (4.6)	10 (3.1)	
Other a	0 (0.0)	27 (8.4)	
Attending seen, *n* (%)	142 (72.8)	183 (57.0)	< .001
Resident seen, *n* (%)	56 (28.7)	62 (19.3)	.01
APC seen, *n* (%)	61 (31.3)	161 (50.2)	< .001
Nurse seen, *n* (%)	131 (67.2)	160 (49.8)	< .001
Concordance with…, *n* (%)			
Attending	94 (48.2)	0 (0.0)	
Resident	34 (17.4)	0 (0.0)	
APC	53 (27.2)	0 (0.0)	
Nurse	109 (55.9)	0 (0.0)	
Number of concordant clinicians, *n* (%)			
0	0 (0.0)	321 (100.0)	
1	114 (58.5)	0 (0.0)	
2	47 (24.1)	0 (0.0)	
3+	34 (17.4)	0 (0.0)	
Rating of ED care, *n* (%)			.19
0	13 (6.7)	16 (5.0)	
1	8 (4.1)	7 (2.2)	
2	13 (6.7)	17 (5.3)	
3	31 (15.9)	46 (14.3)	
4	49 (25.1)	65 (20.3)	
5	81 (41.5)	170 (53.0)	
Binary rating of ED care, *n* (%)			.12
Low rating (0 – 2)	34 (17.4)	40 (12.5)	
High rating (3 – 6)	161 (82.6)	281 (87.5)	
Rating of ED care, median (IQR)	4 [3, 5]	5 [3, 5]	.01
Impact of race on respect^c^, median, (IQR)	0 [0, 2]	0 [0, 3]	.25
Effect of race on quality of service^d^, median (IQR)	0 [0, 0]	0 [0, 1]	.10
Effect of race on communication^e^, median (IQR)	0 [0, 0]	0 [0, 0]	.90
Effect of race on care			.10
Positive	22 (11.3)	23 (7.2)	
Negative	10 (5.1)	29 (9.0)	
None	156 (80.0)	258 (80.4)	
Missing	7 (3.6)	11 (3.4)	

a Including multiracial, Native American or American Indian, Native Hawaiian or Pacific Islander, or self-identified as other or unknown.

b Clinicians seen indicates how many patients within each group saw a given level of clinician. Because some patients saw more than 1 type of clinician, the percentages total greater than 100%.

c Participants were asked, “Did you feel your race affected how much respect you were treated with? From 0–5, 0 = not at all, 5 = strongly affected”. 488 participants completed this question.

d Participants were asked , “Did you feel your race affected the quality of service you experienced? From 0–5, 0 = not at all, 5 = strongly affected”. 468 participants completed this question.

e Participants were asked, “Did you feel your race affected the communication between you and the care team? From 0–5, 0 = not at all, 5 = strongly affected”. 467 participants completed this question.

*APC*, advanced practice clinician; *IQR*, interquartile range; *NCD*, not completely discordant; *SD*, standard deviation.

**Table 2:** Ordinal logistic regression for concordance and effect of race on the primary outcome.

Predictor		Adjusted Odds Ratio (OR)	95% CI		*P* value
			Lower	Upper	
Not complete discordance *(Reference: Complete discordance)*		0.69	0.40	1.17	.17
Perceived effect of race on care *(Reference: No perceived impact)*	Positive	1.21	0.66	2.21	.54
	Negative	0.16	0.09	0.30	< .001
	No response	0.92	0.36	2.32	.85
Female sex *(Reference: Male)*		0.82	0.57	1.19	.30
Race *(Reference: White)*	Black	1.31	0.74	2.31	.35
	Asian	1.26	0..52	3.05	.61
	Other	0.75	0.31	1.84	.53
Age (per year)		1.01	1.00	1.02	.03
