# Supplementary material for: Effects of Racial Concordance and Perceived Racism on Emergency Department Patient Satisfaction
Source: West J Emerg Med. 2026 Oct 26;27(6):1678–83. doi: 10.5811/westjem.49041 (PMC13761928; doi:10.5811/westjem.49041)
Supplement: Supplementary file 1 [file wjem-27-1678-s001.docx]

**Figure S1.** Patient Flow

4,306 patients discharged during the study period

3,548 had listed mobile phone number and were texted

590 provided informed consent (Question 1 of survey)

20 texted back "No"

2,938 did not respond

516 completed experience rating question and were included in analysis (Question 2 of survey)

**Figure S2.** Distribution of Experience Ratings
