# Supplementary material for: Effects of Racial Concordance and Perceived Racism on Emergency Department Patient Satisfaction
Source: West J Emerg Med. 2026 Oct 26;27(6):1678–83. doi: 10.5811/westjem.49041 (PMC13761928; doi:10.5811/westjem.49041)
Supplement: Supplementary file 2 [file wjem-27-1678-s002.docx]

**Table S1.** Stratified univariate analyses of patient race, clinician seen, and perceived impact of race on care on median experience rating

|  | Median rating of ED care (IQR) | | |
| --- | --- | --- | --- |
|  | Not complete discordance | Complete racial discordance | p-value |
| Patient race |  |  |  |
| Black | 4 [3, 5] | 5 [3, 5] | .13 |
| White | 4 [3, 5] | 5 [4, 5] | .06 |
| Asian | 5 [4, 5] | 4 [4, 5] | .51 |
| Clinician Seen |  |  |  |
| Attending | 4 [3, 5] | 5 [3, 5] | .04 |
| Resident | 4 [3, 5] | 5 [4, 5] | .11 |
| APC | 4 [3, 5] | 5 [4, 5] | .09 |
| Nurse | 4 [3, 5] | 4 [3, 5] | .18 |
| Perceived effect of patient race on ED care |  |  |  |
| Positive | 4.5 [4, 5] | 5 [3, 5] | .93 |
| Negative | 3 [2, 4] | 3 [0, 4] | .84 |
| None | 4 [3, 5] | 5 [4, 5] | < .001 |

*ED,* emergency department; *IQR*, interquartile range; *APC;* advanced practice clinician

**Table S2.** Poisson regression with robust standard errors and adjusted relative risk (RR)

| Predictor | | | | | Adjusted RR | Coefficient | Robust Standard Error | 95% CI | | p-value |
| --- | --- | --- | --- | --- | --- | --- | --- | --- | --- | --- |
|  |  |  |  |  |  |  |  | Lower | Upper |  |
| Not complete discordance  (Reference: Complete Discordance) | | | | | 1.00 | -0.00 | 0.05 | -0.10 | 0.10 | > .999 |
| Perceived effect of race on care  (Reference: No perceived impact) | | | Positive | | 0.95 | -0.05 | 0.07 | -0.19 | 0.08 | .44 |
|  |  |  | Negative | | 0.63 | -0.47 | 0.14 | -0.75 | -0.20 | .001 |
|  |  |  | No response | | 0.98 | -0.02 | 0.08 | -0.19 | 0.14 | .80 |
| Female sex (Reference: Male) | | | | | 1.01 | 0.01 | 0.04 | -0.07 | 0.08 | .87 |
| Race (Reference: White) | | | Black | | 1.12 | 0.11 | 0.06 | -0.005 | 0.22 | .06 |
|  |  |  | Asian | | 1.21 | 0.19 | 0.06 | 0.07 | 0.30 | .002 |
|  |  |  | Other | | 1.03 | 0.03 | 0.10 | -0.18 | 0.23 | .80 |
| Age | | | | | 1.00 | 0.001 | 0.001 | -0.001 | 0.003 | .29 |
|  |  |  | | In a multivariable analysis controlling racial concordance, perceived effect of race on care, sex, race, and age as predictors of satisfaction rating, two significant predictors of satisfaction rating were identified. Patients believed race negatively affected the care they received had decreased odds of higher satisfaction ratings. Increased age trended towards an increased odds of higher satisfaction ratings. | | | | | | |

*CI*, confidence interval
